# Soil Microsite Outweighs Cultivar Genotype Contribution to *Brassica* Rhizobacterial Community Structure

**DOI:** 10.3389/fmicb.2021.645784

**Published:** 2021-04-07

**Authors:** Scott A. Klasek, Marcus T. Brock, Hilary G. Morrison, Cynthia Weinig, Loïs Maignien

**Affiliations:** ^1^Marine Biological Laboratory, Josephine Bay Paul Center for Comparative Molecular Biology and Evolution, Woods Hole, MA, United States; ^2^Department of Botany, University of Wyoming, Laramie, WY, United States; ^3^Program in Ecology, University of Wyoming, Laramie, WY, United States; ^4^Department of Molecular Biology, University of Wyoming, Laramie, WY, United States; ^5^UMR 6197, Laboratory of Microbiology of Extreme Environments, Institut Européen de la Mer, Université de Bretagne Occidentale, Brest, France

**Keywords:** rhizosphere, soil microsites, bacterial communities, biomarkers, *Brassica*

## Abstract

Microorganisms residing on root surfaces play a central role in plant development and performance and may promote growth in agricultural settings. Studies have started to uncover the environmental parameters and host interactions governing their assembly. However, soil microbial communities are extremely diverse and heterogeneous, showing strong variations over short spatial scales. Here, we quantify the relative effect of meter-scale variation in soil bacterial community composition among adjacent field microsites, to better understand how microbial communities vary by host plant genotype as well as soil microsite heterogeneity. We used bacterial 16S rDNA amplicon sequencing to compare rhizosphere communities from four *Brassica rapa* cultivars grown in three contiguous field plots (blocks) and evaluated the relative contribution of resident soil communities and host genotypes in determining rhizosphere community structure. We characterize concomitant meter-scale variation in bacterial community structure among soils and rhizospheres and show that this block-scale variability surpasses the influence of host genotype in shaping rhizosphere communities. We identified biomarker amplicon sequence variants (ASVs) associated with bulk soil and rhizosphere habitats, each block, and three of four cultivars. Numbers and percent abundances of block-specific biomarkers in rhizosphere communities far surpassed those from bulk soils. These results highlight the importance of fine-scale variation in the pool of colonizing microorganisms during rhizosphere assembly and demonstrate that microsite variation may constitute a confounding effect while testing biotic and abiotic factors governing rhizosphere community structure.

## Introduction

Plant-associated microbial communities have been referred to as the “extended phenome” of plants, in part due to the pervasive effects they have on host phenotypes (Berendsen et al., 2012; Qu et al., 2020). In particular, microbes can affect host plant growth patterns by increasing plant nutrient access (Chen et al., 2002; Richardson et al., 2009; Richardson and Simpson, 2011), relieving abiotic stress (Zolla et al., 2013; Timmusk et al., 2014), and by reducing susceptibility to biotic stressors such as herbivores (Pineda et al., 2010; Hubbard et al., 2019) and pathogens (Mendes et al., 2011, 2013; Durán et al., 2018). Characterizing and potentially manipulating the factors that determine rhizosphere microbial community assembly will likely contribute to future sustainable agriculture and land management practices (Busby et al., 2017; Ke et al., 2020).

For plants growing in agricultural settings and natural populations, host genotypes (Fitzpatrick et al., 2018) and soil heterogeneity (Mapelli et al., 2018; Zhou et al., 2020) are critical factors contributing to the assembly of rhizosphere communities. Though host genotype may explain a small amount (2–3%) of variance within rhizosphere communities in natural *Arabidopsis thaliana* (Bulgarelli et al., 2012; Lundberg et al., 2012; Thiergart et al., 2020) and wheat cultivars (Simonin et al., 2020), field studies in maize have identified marker gene sequences that repeatedly associate with specific genotypes over the scale of years (Walters et al., 2018). Plant species composition has been shown to explain variation in soil microbial communities that cannot be accounted for by edaphic factors alone (Leff et al., 2018). Artificial selection of crop species can also elicit dramatic morphological and physiological shifts likely to influence host–microbe interactions (Pérez-Jaramillo et al., 2016), but few studies have examined rhizosphere bacterial (hereafter, rhizobacterial) community variation within highly phenotypically differentiated crop varieties of a species (Marques et al., 2014). In addition to host plant genotype, soil environmental heterogeneity can also influence microbial community structure. Plant genotype and field site accounted for nearly half of the total variance among rhizosphere communities from 26 inbred maize genotypes grown in five field sites across the midwest and northeast United States, though the authors noted field-specific variability in bacterial relative abundance enriched among certain genotypes (Peiffer et al., 2013). Rhizobacterial communities are largely influenced by soil communities they recruit from Vieira et al. (2020), and soil spatial heterogeneity at the centimeter-scale can contribute to variation in relative abundance of bacterial phyla (O’Brien et al., 2016) and even outweigh seasonal changes at scales of meters (Carini et al., 2020).

**FIGURE 1:**
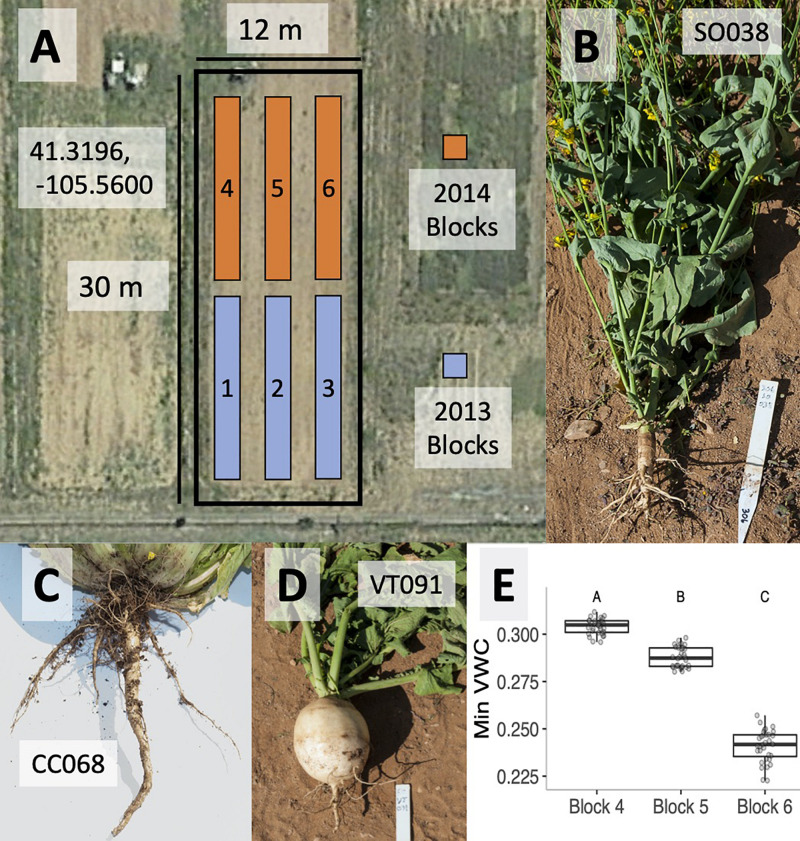
Satellite photograph of the field site in Laramie WY (centered on 41.3196 N, 105.56 W) showing the three blocks used for each growing season **(A)**. Root architecture photographs for cultivars of summer oilseed **(B)**, Chinese cabbage **(C)**, and vegetable turnip **(D)**. BB (weed) crops physically resemble summer oilseeds. Variation in average minimum volumetric water content (VWC) across blocks 4–6 in the 2014 experiments is depicted. Letters denote significantly different block effects via a *post hoc* Tukey’s test **(E)**.

Here, we investigate the relative contributions of host plant genotype and field microsite heterogeneity on bacterial community assembly in rhizospheres of four *Brassica rapa* cultivars. *B. rapa* does not form symbiotic associations with arbuscular mycorrhizal fungi (Delaux et al., 2014) and is characterized by dramatic changes in root morphology: varieties include cultivated turnips (subspp. *rapa*) with large root-like storage organs, as well as cabbages (subspp. *chinensis* and *pekinensis*) and oilseeds (subspp. *oleifera*) that have greater masses of secondary roots (Cheng et al., 2016). Brassicaceous crop types also differ significantly in carbon fixation (Edwards et al., 2011; Yarkhunova et al., 2016), which may affect the quality or quantity of root exudates that, in turn, can influence rhizobacterial community assembly. To encompass genetic variation within *B. rapa*, we selected a single representative genotype from each of the three major crop types (turnip, seed oil, and Chinese cabbage) as well as a weedy accession collected from California, United States (see Supplementary Table 1 for accession details). These genotypes were grown in six field blocks across 2 years to evaluate the relative contribution of host plant genotype vs. agricultural microsite (i.e., site distances ranging several meters, Figure 1A) on rhizobacterial communities. Though we expected host genotype to influence rhizobacterial community structure to a higher degree than soil microsite, we identified high numbers of amplicon sequence variants (ASVs) differentially abundant across each microsite and found that microsite played a larger influence than genotype in this agricultural system.

## Materials and Methods

### Field Experiments

In order to explore the effects of plant genotype and microsite on rhizobacterial community composition, we raised *B. rapa* genotypes during the summers of 2013 and 2014 in a randomized block design. Blocks corresponding to each year are shown in Figure 1A. Soil and rhizosphere samples were collected from agricultural fields at the University of Wyoming (Laramie, WY, United States; 41.3198 N, −105.5598 W; 2217 m). Crop cover history of this field has predominantly consisted of *B. rapa*, *Brassica napus*, and *A. thaliana* since 2010; *B. rapa* crop types were grown in blocks 1–3 in 2012, while *A. thaliana* crop types were grown in blocks 4–6 in the 2013 growing season. Soil was tilled each season in the spring and fall. A routine soil analysis from field soil in 2014 (Colorado State University Extension) estimated the texture as a slightly basic sandy clay loam, with a pH of 7.9 and a 2.1% organic matter (Supplementary Table 2).

#### Field Season 2013

On June 6, 2013, we planted three to four surface-sterilized seeds of each of four genotypes into five randomly assigned sites in each of three spatial blocks (Figure 1A). Planting sites were arranged in a rectangular grid of four columns with 25 cm between neighboring plants. In addition, five sites in each block were left unplanted in order to characterize the bulk soil microbial community. One to three replicates per genotype per block were harvested between September 18 and 20, 2013, in order to characterize the *B. rapa* rhizosphere microbial community using methods identical to the larger 2014 experiment described below.

#### Field Season 2014

On June 13, 2014, we planted surface-sterilized seeds of each of four genotypes into seven randomly assigned cells in each of three spatial blocks (Figure 1A). Each block consisted of 40 planting sites (4 columns × 10 rows) with 25 cm between neighboring plants. Ten of the unvegetated cells in each block were assigned to the bulk soil treatment. A day before planting, the agricultural fields were tilled to homogenize the soil microbial community and facilitate planting. Using gloves sterilized with 70% EtOH, surface-sterilized seeds were placed in small depressions at each planting site and topped with 250 ml of sterilized Redi-Earth potting soil (Sun Gro Horticulture, Agawam, MA, United States) and vermiculite (50:50 by volume) to secure seed placement and to enhance water availability during germination. Seeds were surface-sterilized by vortexing in 70% EtOH (1 min) and 10% bleach (10 min) followed by four rinses in autoclaved RO water. The soil mix was sterilized via 2 × 1-h autoclave cycles. Blocks were irrigated twice daily (04:30 and 18:00 h), and each planting site was thinned to one seedling 2 weeks following germination.

In each block, we monitored soil temperature, soil moisture (volumetric water content; VWC; Decagon 5TE sensor; Decagon Devices, Pullman, WA, United States), ambient temperature, and relative humidity (EHT Temperature/RH sensor, Decagon Devices, Pullman, WA, United States) at continuously running 5 min averages to determine if any of these abiotic factors co-varied with block-associated microbial community composition. We estimated mean day and night abiotic values for each block, and in order to limit temporal autocorrelation, we randomly selected 16 days with which to test for abiotic differences across blocks using ANOVA. Of these parameters, only VWC varied significantly between blocks (Figure 1).

### Soil and Rhizosphere Sampling

Soils from five randomly selected unvegetated plots in each block were sampled on July 8, 2014, after 2 weeks of growth, and again during harvesting (8/19/14 to 8/22/14). At each of the five sites, we used an ethanol-sterilized trowel to collect a 15 cm deep soil sample from which we subsampled visually equivalent small amounts of soil from the top (0–5 cm), middle (5–10 cm), and bottom (10–15 cm) horizons using a sterile spatula. The three subsamples were pooled into a single sterile 50 ml tube and placed on ice until processing with PBS buffer in the laboratory later that day. Pooling samples allowed us to capture the unvegetated site variation in microbial community composition found across the 15 cm “trowel-depth” soil profile from which *Brassica* roots were harvested. In August, we randomly selected five replicates of each genotype in each block to harvest for rhizosphere microbial communities. Because only two replicates of the seed oil survived in block 6, we harvested two additional replicates of this genotype from block 4. Over 4 days (8/19/14 to 8/22/14; 12:00 h), we harvested *Brassica* rhizospheres by digging up plants with a trowel and shaking off bulk soil from the root system. In order to target the rhizosphere of each plant (as defined by soil <2 mm from the primary or secondary roots at ≤15 cm depth), we more carefully removed larger soil clumps with a metal spatula. Root systems were cut, placed in a sterile sample bag, and then placed on ice until further processing. All tools were cleaned using 70% EtOH, and gloves were changed before processing the next sample.

In the lab, 40 ml of PBS buffer with 0.02% Silwet L-77 (PBSS) was added to each bag, shaken vigorously for 1 min, and transferred to a sterile 50 ml centrifuge tube. Tubes were vortexed for 5 min, and samples were filtered through a 100 μm nylon mesh Steriflip unit (Millipore Sigma, Burlington, MA, United States) to remove larger soil particles and fine roots. Tubes were centrifuged at 3,500 rpm for 15 min at 10°C to pellet the microbial community and the supernatant was removed. Pellets were then re-suspended in PBSS (volume based on pellet mass) and a volume equivalent to 200 mg of pellet was transferred to a new microcentrifuge. After centrifuging once more (3200 RCF for 15 min at 10°C), the supernatant was removed, and tubes were snap-frozen in liquid nitrogen and stored at −80°C for DNA extraction.

### DNA Extraction, 16S Amplification, and Sequencing

Rhizosphere DNA was isolated from ∼0.2 g of pelleted rhizosphere soil using the PowerLyzer PowerSoil Kit (Qiagen, Hilden, Germany) according to manufacturer instructions and stored at −80°C. Amplicon libraries were constructed and sequenced at the Josephine Bay Paul Center at the Marine Biological Laboratory, following the protocol described in Eren et al. (2013). Briefly, we used the pooled, unfused 16S 967F primers (CTAACCGANGAACCTYACC, CNACGCGA AGAACCTTANC, CAACGCGMARAACCTTACC, and ATAC GCGARGAACCTTACC) and 1064R (CGACRRCCATGCAN CACCT) to amplify the V6 hypervariable region of the bacterial 16S rRNA gene. PCR was performed using Platinum HiFi Taq polymerase (Thermo Fisher Scientific, Waltham, MA, United States), starting with 3 min at 94°C, followed by 25 cycles of 30 s at 94°C, 45 s at 60°C, and 1 min at 72°C, and followed by an elongation step of 2 min at 72°C. Undesired small products were removed using 96-well MinElute plates (Qiagen, Hilden, Germany). The first round of PCR products were eluted in 30 μl of Qiagen buffer EB, and 4 μl of the eluate was used in a 5-cycle amplification with barcoded and indexed fusion primers that contained 5′ Illumina-specific adapters permitting flow cell binding and sequencing. Double-stranded DNA was quantified with PicoGreen (Thermo Fisher Scientific, Waltham, MA, United States), pooled in equimolar amounts, and size selected using a PippinPrep (Sage Science, Beverly, MA, United States). Sequencing was conducted on an Illumina HiSeq 1000 (2013 datasets) or a NextSeq 500 (2014 datasets).

### Sequence Processing and Analysis

For both 2013 and 2014 datasets, Trimmomatic version 0.36 (Bolger et al., 2014) was used to remove adapters, primer sequences, and reverse complements of R2 primers from forward (R1) reads of 16S rRNA V6 sequences. Working in R version 3.6.2, we used DADA2 version 1.14.0 (Callahan et al., 2016) to denoise raw reads into amplicon sequence variants (ASVs) from forward reads and remove chimeric sequences. We used version 132 of the SILVA non-redundant 16S reference database (Quast et al., 2013) to assign taxonomies to ASVs. Sequences were aligned with DECIPHER version 2.14.0 (Wright et al., 2012), and phylogenetic trees were generated with Phangorn 2.5.5 (Schliep, 2011). For both datasets, Phyloseq version 1.30.0 (McMurdie and Holmes, 2013) was used to combine sample information, count and taxonomy tables, and phylogenetic trees into a single object for subsequent interactive analyses in R. Sequences identified as eukaryotes, chloroplasts, mitochondria, or belonging to unclassified domains were removed from both datasets. The presence of one PCR blank and six DNA extraction blanks in the 2014 dataset permitted identification of contaminant sequences with decontam version 1.6.0 (Davis et al., 2018). In addition, PicoGreen quantitation of amplicon libraries allowed us to identify contaminants based on higher relative abundances in blanks. In total, we identified and removed 63 contaminant ASVs from the 2014 dataset: 29 by the decontam prevalence method using a *p*-value of 0.5, and 40 using the combined method (six ASVs were identified by both methods). After identifying contaminants, blanks were removed from the phyloseq object, leaving a minimum library size of 19,421 reads. All communities from the 2013 dataset were more deeply sequenced than those from 2014, with median read numbers totaling 626,560 compared to 115,806. A lack of blanks and amplicon concentration data prohibited decontamination in the 2013 dataset, though 30 contaminants from 2014 appeared in the 2013 dataset and accounted for no more than 0.23% of reads in any community. ASV read count tables were transformed using Hellinger, cumulative sum scaling, and variance-stabilizing methods from vegan version 2.5–6 (Oksanen et al., 2008), metagenomeSeq version 1.29.1 (Paulson et al., 2013), and DESeq2 version 1.26.0 (Love et al., 2014), respectively. Cumulative sum scaling captured slightly more variance than the other normalization methods (data not shown), so this method was used to generate final ordinations. Ordinations and PERMANOVA tests were conducted with vegan, and DESeq2 was used to determine biomarker ASVs for all sample categories. Reproducible code for all analyses will be made freely available upon acceptance on Github at https://github.com/loimai/bloc_crop_16S_V6). Raw 16S sequence data were deposited to NCBI under the bioproject number PRJNA668184.

## Results

### Field Experiments

In 2013, we raised plants primarily to determine rhizosphere and bulk-soil collection protocols. Small sample sizes and loss of plants due to mortality during that summer prohibited us from robustly testing the effect of genotype × block interaction on microbial communities. Here, we focus predominantly on rhizosphere and bulk-soil collections from 2014, where we had sufficient sampling within and across blocks to test how genotype, block, and the genotype × block interaction impacted rhizosphere microbial communities. Microbial samples from 2013, which were collected using the same methods as in 2014, are included to examine patterns in community structure and biomarker distributions across years.

Variable root architecture among *B. rapa* cultivars is seen in summer oilseed (SO038, Figure 1B), Chinese cabbage (CC068, Figure 1C), and vegetable turnip (VT091, Figure 1D). Root morphology of the Back Bay weedy accession (BB) was more similar to that of the summer oilseed cultivar. Soil temperature, ambient temperature, and relative humidity did not vary significantly between blocks in the 2014 experiment, though mean minimum volumetric water content was highest in block 4 and lowest in block 6 (Figure 1E; ANOVA *p* < 0.0001).

### Patterns of Bacterial Community Composition and Alpha Diversity

We found 21,341 ASVs in 2013 and 13,220 in 2014, reflective of the higher sequencing depth in the earlier dataset; 60–65% of the 16S V6 reads could be assigned to phylum, 17–20% to genus, and 1.4% to species. Composition barplots of rhizosphere and soil bacterial communities are shown in Supplementary Figures 1, 2. The 1000 most abundant ASVs across each dataset accounted for approximately 75–80% of communities, and the top 100 ASVs for 30–40%. Communities were generally dominated by two phyla (Actinobacteria and Proteobacteria), which were more dominant in soils and rhizospheres, respectively. At the Class level, communities from 2014 were characterized by lower relative abundances of Bacilli and higher proportions of Gammaproteobacteria in comparisons to those from 2013 (Supplementary Figures 1, 2). Measurements of richness (observed ASVs) and evenness (inverse Simpson and Shannon indices) in bulk soil communities were no higher than in rhizospheres (Supplementary Figure 3). On a few occasions, these estimates of alpha diversity seemed to vary by block and genotype, though greater numbers of samples would be required to determine statistical significance.

**FIGURE 2:**
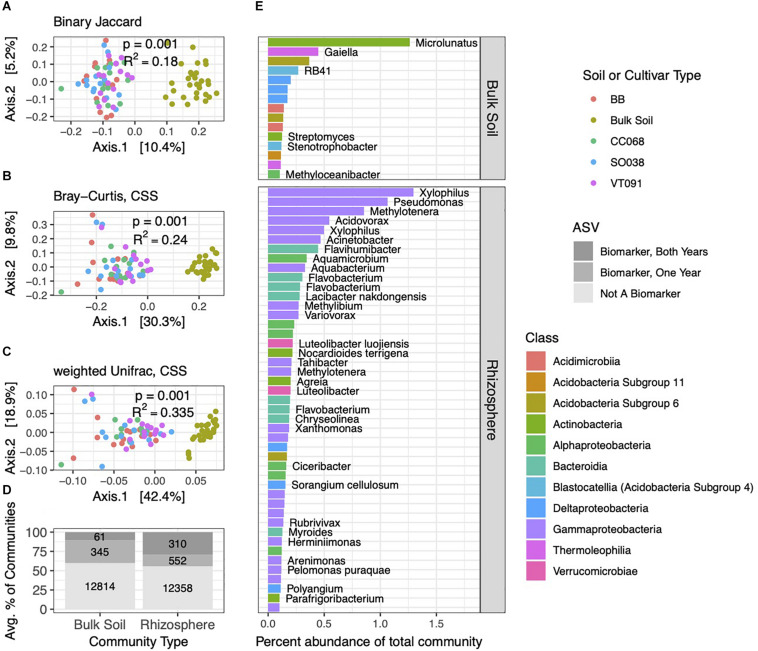
*Brassica* rhizospheres and surrounding soils show distinct bacterial community structures. PCoA ordinations of binary Jaccard **(A)**, Bray–Curtis **(B)**, and weighted Unifrac **(C)** distance metrics from 2014 collections. PERMANOVA tests reveal factors correlated with soil and rhizosphere community structure, with *p* and *R*^2^ values inlaid within ordinations. **(D)** Numbers and average percent abundances of ASVs identified as biomarkers that correspond to rhizospheres and soils. Sets of biomarkers for these groups found in both 2013 and 2014 datasets are colored in darkest gray; progressively lighter shades show biomarkers identified in one of the two years, and non-biomarkers. **(E)** The most abundant bacterial ASVs (>0.1% average abundance in soil or rhizosphere communities) of these biomarker sets, with genus and species names, where identified.

### Bacterial Community Composition in the Rhizosphere and Bulk Soil Is Distinct and Repeatable Across Seasons

The bacterial community structures in 2014 and 2013 experiments showed clear separations between soil and rhizosphere habitats, with little discernible difference between rhizospheres of different plant genotypes (Figures 2A–C). Compared to the binary Jaccard distance metric, Bray–Curtis and weighted Unifrac captured increasing variance on the two principal coordinate analysis (PCoA) primary axes, revealing the combined influences of relative abundance and phylogeny in differentiating these two habitats.

We next evaluated which ASVs were significantly differentially distributed between soil and rhizosphere communities (which we hereafter refer to as biomarkers). Among 13,220 ASVs from the 2014 dataset, we identified 862 rhizosphere and 406 soil biomarkers, which made up a sizable minority of the community by percent abundance (Figure 2D). A subset of these was also detected as biomarkers in the 2013 dataset. Of these both-year biomarkers, 310 rhizosphere biomarkers comprised a higher percentage of rhizosphere communities than the 61 soil biomarkers. Across both habitats, sets of biomarker ASVs included the more abundant community members overall, and this pattern was most pronounced in rhizosphere biomarkers identified in both years (Figure 2D). Distributions of biomarkers across both habitats in 2013 roughly mirrored 2014 (Supplementary Figure 4). Several genera of Gammaproteobacteria, such as *Xylophilus*, and Bacteroidia, such as *Flavobacterium*, ranked among the most abundant repeat-season rhizosphere biomarkers, while repeat-season soil biomarkers were more evenly distributed across several classes (Figure 2E).

### Soil and Rhizosphere Bacterial Communities Vary Across Blocks

**FIGURE 3:**
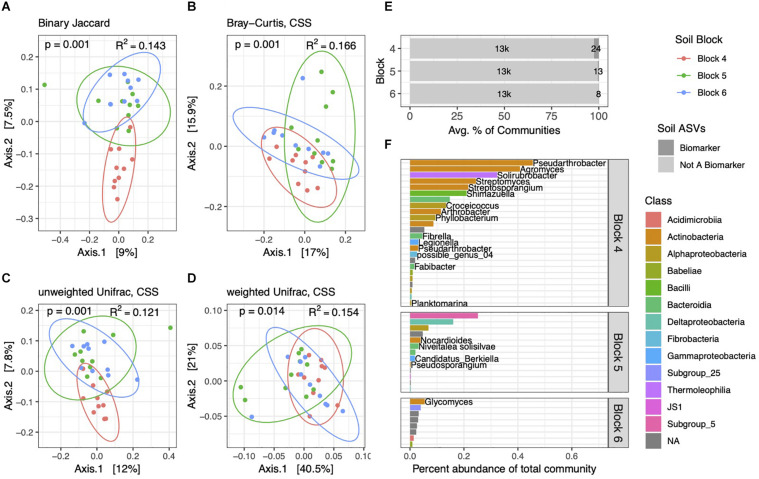
Soil bacterial community structure varies by block. PCoA ordinations of soil community structure using binary Jaccard **(A)**, Bray–Curtis **(B)**, unweighted Unifrac **(C),** and weighted Unifrac **(D)** distance metrics. PERMANOVA tests reveal differences between blocks, with *p* and *R*^2^ values inlaid within ordinations. **(E)** Biomarker ASVs specific to each block represent small but variable percent abundances of soil communities. **(F)** Average percent abundances of all biomarker ASVs within their respective blocks are shown, as well as genus and species names when identified. All data are from the 2014 dataset.

Having characterized differences between bulk soil and rhizosphere bacterial communities, we next examined soil community differences across blocks, focusing only on the 2014 dataset. Though blocks were spatially separated by less than 20 m (Figure 1A), block-specific differences between soil bacterial communities were apparent across all distance metrics that we tested (Figures 3A–D). Ordinations with different distance metrics again showed relative abundance and phylogenetic information explaining increasing amounts of variance in soil community structure.

Though follow-up pairwise PERMANOVA tests with binary Jaccard, Bray–Curtis, and unweighted Unifrac distances showed each block to be distinct from one another, weighted Unifrac tests showed that block 6 was not significantly different from block 4 (*p* = 0.132) or from block 5 (*p* = 0.114), though blocks 4 and 5 were statistically different (*p* = 0.001). This is bolstered by our observation that block 6 soil communities had fewer numbers and lower percent abundances of block-specific biomarkers than the other two (Figure 3E). In contrast to our prior comparisons between soils and rhizospheres, we identified only 45 ASVs in soil communities that differed across blocks, and these block-specific biomarkers comprised 0.23–2.7% of communities. Many of the most abundant block biomarkers in soil communities, particularly in block 4, consisted of Actinobacteria (Figure 3F), while several others, including many of the lower abundance ASVs, could not be identified at Genus or even Class levels.

**FIGURE 4:**
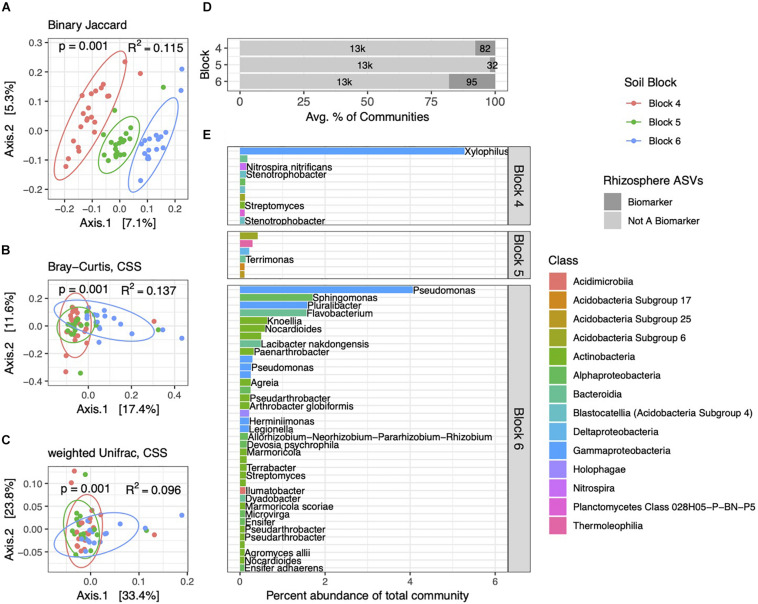
Rhizosphere bacterial community structure varies by block. PCoA ordinations of rhizosphere communities showing binary Jaccard **(A)**, Bray–Curtis **(B)**, and weighted Unifrac **(C)** distance metrics. Communities are distinct among the three blocks, as indicated by *p* and *R*^2^ values. **(D)** Total numbers and percent abundances of biomarker ASVs specific to each block represent small but variable percentages of rhizosphere communities. **(E)** Average percent abundances of the most abundant (>0.1%) rhizosphere biomarker ASVs within their respective blocks are shown, as well as genus and species names when identified. All data are from 2014.

As with soils, bacterial community structure in rhizospheres varied across the three blocks, with Bray–Curtis and weighted Unifrac distance metrics capturing increasing variation (Figures 4A–C). Interestingly, the PCoA plot derived from the binary Jaccard distance (Figure 4A) shows the most distinct clustering of communities by block, suggesting that the presence or absence of certain ASVs drives these block-specific differences in rhizosphere communities more noticeably than in soils. Block-specific biomarker ASVs made up variable but generally higher proportions of rhizosphere communities (2–18%) than in soils (Figure 4D), a trend we also observed in the 2013 dataset (Supplementary Figure 4). The most abundant block biomarkers in rhizobacterial communities were Gammaproteobacteria, notably ASVs from the genera *Xylophilus* and *Pseudomonas* (Figure 4E). Some genera contained multiple biomarkers specific to certain blocks (*Nocardioides* and *Marmoricola* within block 6), while others (*Pseudarthrobacter*, *Streptomyces*, *Legionella*, and *Halangium*) contained biomarker ASVs specific to different blocks.

### Block Biomarkers Common to Rhizospheres and Soils Are Low Abundance in Both Habitats

**FIGURE 5:**
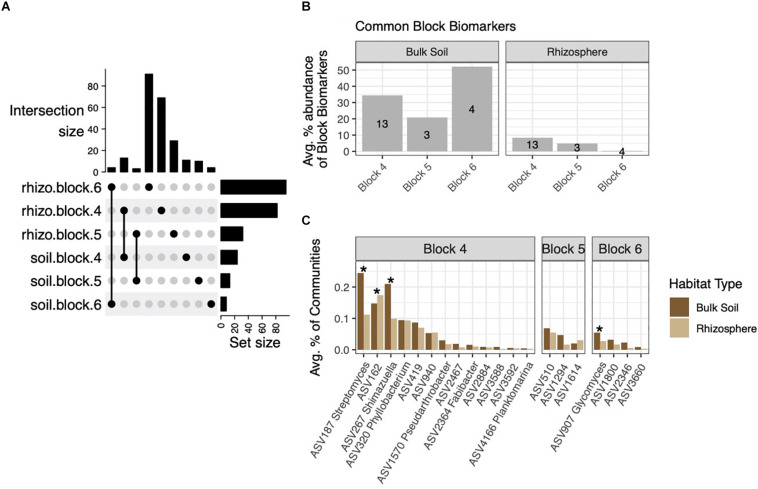
The intersection of soil and rhizosphere block biomarkers. **(A)** Upset plot showing biomarkers from each block that are common to both rhizosphere and soil habitats. **(B)** These common block biomarkers make up a small percentage of rhizosphere block biomarkers, but a larger fraction of soil block biomarkers. **(C)** Average percent abundances of common block biomarker ASVs within their respective blocks across both habitats, with ASVs differentially abundant across habitats noted by asterisks (adjusted *p* < 0.05).

Having identified block-specific biomarkers in soil and rhizosphere habitats, we next investigated how many of the rhizosphere block biomarkers were derived from soil block biomarkers. These six sets of ASVs overlapped only between habitats from the same block (Figure 5A). Due to the high numbers of rhizosphere block biomarkers, block biomarkers common to both rhizospheres and soils made up a much smaller percentage of total block biomarkers in rhizospheres than in soils: These common biomarkers comprised 21–52% of soil block biomarkers (Figure 5B). On average, common block biomarkers made up no more than 0.25% of rhizosphere or soil communities, and were generally evenly distributed between the two habitats or more highly abundant within soils (Figure 5C). Variable distribution of these biomarkers across the three blocks led us to suspect the influence of soil community structure on rhizospheres may differ at scales of several meters. To address this, we used FEAST, a source-tracking method that estimates proportions of source microbial communities (in our case, soils) on sink (rhizosphere) communities (Shenhav et al., 2019). On average, 71.8% of the rhizosphere community composition could be sourced from soil communities (Supplementary Figure 5). When considering each block independently, the percentage was smaller, averaging 61.6%. No difference in source percentages was observed across blocks (*p* = 0.154, *F* = 1.938, one-way ANOVA) (Supplementary Figure 5).

### Rhizobacterial Communities Vary Subtly by Plant Genotype

**FIGURE 6:**
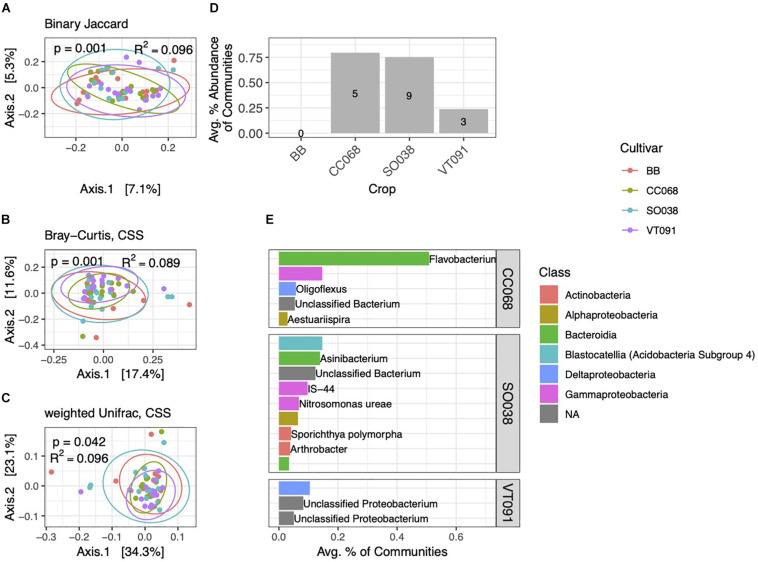
Cultivar-specific variation in rhizosphere bacterial community structure. PCoA ordinations of binary Jaccard **(A)**, Bray–Curtis **(B)**, and weighted Unifrac **(C)** distance metrics explain increasing amounts of variation in rhizosphere community structure. **(D)** Total numbers and percent abundances of biomarker ASVs specific to each cultivar. No biomarkers were detected for BB. **(E)** Average percent abundances of the most abundant (>0.1%) rhizosphere biomarker ASVs for each cultivar are shown, with genus and species names when identified. All data are from 2014.

As among blocks, rhizobacterial community composition also varied among the four *B. rapa* genotypes grown (Figures 6A–C). Genotype alone explained 8.9–9.6% of community variation, whereas block accounted for equal or slightly higher amounts (9.6–13.7%). We note no interaction effect between genotype and block (*p* = 0.334, PERMANOVA on weighted Unifrac distance). In contrast to block-specific differences, PCoA ordinations of communities from different cultivars cluster around a common centroid. In Bray–Curtis and weighted Unifrac ordinations, beta-diversity is highest in BB and SO038 genotypes, and lower in CC068 and VT091 (Figures 6B,C). We also identified far fewer numbers of genotype-specific biomarkers, including none in BB. These biomarkers cumulatively amounted to less than 1% of communities on average (Figure 6D). Higher numbers and relative abundances of biomarkers in communities from CC068 and SO038 genotypes were also seen in the 2013 dataset (Supplementary Figure 4). An ASV belonging to the genus *Flavobacterium* associated with CC068 was the most abundant genotype-specific biomarker (Figure 6E); this was one of the four Flavobacteria enriched in rhizosphere habitats.

## Discussion

### Bulk Soil to Rhizosphere Community Changes

As anticipated, we found a strong rhizosphere effect, noting stark differences between rhizosphere and bulk soil bacterial communities, as reviewed in Bulgarelli et al. (2013). Dominant phyla, including Proteobacteria, Acidobacteria, and Actinobacteria, have been previously identified in *A. thaliana, B. rapa*, and *B. napus* rhizospheres (Bulgarelli et al., 2012; Gkarmiri et al., 2017; Lebreton et al., 2019). Of several core rhizobacterial genera present in the oilseed *B. napus* (Taye et al., 2020), we identified *Arthrobacter* and *Bradyrhizobium* in all 2014 samples, and *Stenotrophomonas* and *Skermanella* in 91 and 74%, respectively. Strains of *Arthrobacter* isolated from rhizospheres can solubilize phosphate (Banerjee et al., 2010) and upregulate salt stress tolerance genes in hosts to promote growth (Khan et al., 2019). *Bradyrhizobium*, commonly associated with root nodulation and nitrogen fixation in legumes, is a genetically diverse genus that can also promote growth in non-leguminous plants such as rice (Piromyou et al., 2015; Ormeño-Orrillo and Martínez-Romero, 2019). Our observation of rhizosphere communities having higher proportions of repeat-year biomarkers than soils may be bolstered by Goss-Souza et al. (2020), who found that tropical forest soil community assembly fits a neutral model in contrast to soybean rhizospheres, which fit a niche-based model.

### Block-Specific Variation

Soil microbial community assembly is thought to have some stochastic influence (Dini-Andreote et al., 2014), though a litany of biotic and abiotic factors can determine taxonomy and function to varying degrees (Fierer, 2017). We would likely require higher replication and a larger dataset containing edaphic, climatological, and/or plant cover information (Sayer et al., 2017) paired with soil and rhizosphere community samples to uncover mechanisms that determine microsite heterogeneity among soil communities. Bulk soil physiochemical measurements from 2014, particularly pH and organic content (Supplementary Table 2), characterize this soil as typical of semiarid agricultural soils from the western United States (Turner et al., 2003). Nevertheless, we identified variability in VWC across blocks 4–6, which may potentially explain high numbers of biomarkers belonging to Actinobacteria in the wettest block (Figure 3F), as observed elsewhere (Liu et al., 2017; Fitzpatrick et al., 2018) and among communities at global scales (Serna-Chavez et al., 2013). Mild drought could also indirectly affect community composition through redox-associated changes, or by influencing plant performance and physiology (Greenham et al., 2017) in ways that alter root exudate profiles (Williams and de Vries, 2020). Block-specific differences in soil community structure are sensitive to distance metrics used, particularly with unweighted metrics showing wetter block 4 samples as noticeable outliers from the others (Figures 3A,C), which suggests that differences between communities from block 4 and the others are most apparent in their composition of rare ASVs. Whether these ASVs represent distinct species or subspecies cannot be resolved with amplicon sequencing methods (Johnson et al., 2019), but meter-scale spatial variation in genetic similarity among populations of grassland soil bacteria has been attributed to gene-specific selection and recombination (Crits-Christoph et al., 2020) that could potentially account for microsite community differences we observe here.

Several studies have noted distinct influences of soil type or soil chemistry on rhizobacterial assembly (de Ridder-Duine et al., 2005; Peiffer et al., 2013; Lagos et al., 2014; Schreiter et al., 2014; Na et al., 2018), and a recent multidisciplinary approach determined that soil communities influenced rhizobacterial composition to a significantly higher degree than plant species, root exudates, and other plant-specific factors (Vieira et al., 2020). The extent to which soil community heterogeneity at small spatial scales (of meters or less) influences rhizosphere assembly remains largely uncharacterized, though our findings that rhizosphere communities were more strongly influenced by soil microsite than by host genotype, and that the majority of rhizosphere communities were sourced from soils (Supplementary Figure 5) are consistent with this observation. Similarly, in *B. napus*, several core rhizobacterial genera were recently identified across field sites and separate growing seasons, though cultivar-specific sequences accounted for some of the community variation (Taye et al., 2020). Within our three blocks, the high variability in numbers and relative abundances of rhizosphere biomarkers (Figure 4D) and the low overlap between these and the set of soil block biomarkers (Figure 5) could reflect differential recruitment strategies that vary at small scales. Block-specific differences in VWC, or other characteristics that we did not measure, could reflect stress responses that may trigger the differential recruitment of rhizosphere bacteria by the host (Santos-Medellín et al., 2017; Naylor and Coleman-Derr, 2018). Our observation of a dominant *Pseudomonas* ASV (4% relative abundance) associated with block 6 rhizospheres (Figure 4E) may hint at a drought stress response, as this block was noticeably lower in volumetric water content than the others (Figure 1E) and members of this genus are well-characterized plant growth promoters capable of alleviating drought stress in a variety of crops (Sandhya et al., 2010; Sarma and Saikia, 2014). Higher organic carbon content, microsite heterogeneity, or root exudate diversity (Zhalnina et al., 2018) may all support a higher diversity of block-specific sequences within rhizospheres as opposed to soils, even though we did not observe higher overall community diversity within rhizosphere samples (Supplementary Figure 3).

Our finding that less than 10% (by abundance) of block biomarkers in rhizospheres were also classified as soil block biomarkers (Figure 5B) is likely a consequence of detecting higher numbers of block biomarkers in rhizosphere habitats. Common block biomarkers being equally distributed across habitats (or higher in soils) suggest that they are more likely opportunistic soil bacteria that may be able to tolerate rhizosphere conditions, rather than relic rhizosphere bacteria from previously grown crops. Prior crop cover has been shown to influence rhizobacterial composition to an extent, though this effect may be species-dependent, as Hilton et al. (2018) did not observe any influence on *B. rapa* rhizosphere communities. Though cultivars of *B. rapa*, *B. napus*, and *Arabidopsis* sp. have been grown at this field site since 2010, densities of Brassicaceous plants from experiments in previous years were low across arable space, and annual spring and fall tilling should have diminished previous crop-cover effects on the microbial community of rhizospheres described here.

We found that *B. rapa* genotype accounted for 8–9% of rhizobacterial community variation (Figures 6A–C), higher than among genotypes of *A. thaliana* (2.4%) (Thiergart et al., 2020) and maize (5–7%) (Peiffer et al., 2013), though considerably lower than the 17% described across 30 species of angiosperms (Fitzpatrick et al., 2018). These PERMANOVA results are given here as a comparison, as this non-parametric method does not account for true variance partitioning. Differences in bacterial relative abundance, and to a lesser extent, composition, appear to distinguish rhizobacterial communities among genotypes (Figures 6A–C). Though host genetic similarity at or below the species level explains little to no variation in rhizobacterial community composition within *Brassicaceae* (Schlaeppi et al., 2014) or angiosperms more generally (Fitzpatrick et al., 2018), patterns have recently been reported within genetic groups of maize (*Zea mays*) (Brisson et al., 2019) and genotypes of oilseeds (*B. napus*) (Taye et al., 2020). More frequently, individual bacterial or fungal sequences that vary significantly in relative abundance among plant genotypes are identified, even when community-wide patterns are not significant (Walters et al., 2018). This subtle variation may be consequential: some strains of *Flavobacteria*, a genus we identified in rhizosphere communities and particularly within CC068 (Figure 6E), are capable of deaminating the stress hormone precursor 1-aminocyclopropane-1-carboxylate deaminase (Madhaiyan et al., 2010) which may alleviate salt stress and promote growth (Glick, 2014; Gupta and Pandey, 2019). Oilseed cultivars of *B. rapa* show higher carbon assimilation rates and stomatal densities compared to vegetable cultivars (Yarkhunova et al., 2016); thus, morphological and metabolic differences may be responsible for variation in nutrient acquisition capability (Zhang et al., 2011) or root exudation profiles that could structure differences in rhizosphere microbial communities between members of highly differentiated plant species. Interestingly, we note that the highest numbers of biomarkers were associated with the oilseed genotype SO038 in both years (Figure 6D and Supplementary Figure 4). Though specific root exudates have been identified in shaping the rhizosphere microbiome in the field (Hu et al., 2018; Zhalnina et al., 2018) and the laboratory (Voges et al., 2019), additional studies should examine how genotype-level differences in root exudate diversity or composition affect rhizosphere community structure and activity.

Our characterization of spatial heterogeneity in bacterial communities within agricultural soils and rhizospheres reveals that variation among plots at meter-scales can outweigh the impact of plant genotypes in shaping rhizobacterial communities. Exact sequence variants were found to associate with each microsite and most host genotypes in repeatable ways across two growing seasons. The low intersection of soil- and rhizosphere-specific biomarkers within blocks reinforces the notion that these habitats are distinguished by differential selection pressures, whose mechanisms require further study in characterizing these heterogeneous environments. Finally, our results have important implications for future experimental designs involving plant growth in the field. Spatial effects identified here can become confounding factors when testing the influence of given parameters on rhizosphere community structure, especially when these effects are subtle, such as the plant genotypes tested in this study. We posit that large numbers of replicates with randomized and balanced designs should be considered to account for this microsite effect.

## Data Availability Statement

Reproducible code for all analyses are freely available as an archived Github repository at https://loimai.github.io/bloc_crop_16S_V6/. Raw 16S sequence data were deposited to NCBI under the bioproject number PRJNA668184.

## Author Contributions

MB, CW, and LM designed the study. MB conducted the fieldwork. HM was responsible for DNA sequencing. SK conducted the data analyses and wrote the manuscript with assistance from MB, CW, HM, and LM. All authors contributed to the article and approved the submitted version.

## Conflict of Interest

The authors declare that the research was conducted in the absence of any commercial or financial relationships that could be construed as a potential conflict of interest.
