# Supplementary material for: Soil Microsite Outweighs Cultivar Genotype Contribution to Brassica Rhizobacterial Community Structure
Source: Front Microbiol. 2021 Apr 7;12:645784. doi: 10.3389/fmicb.2021.645784 (PMC8058099; doi:10.3389/fmicb.2021.645784)
Supplement: Supplementary file 2 [file Data_Sheet_2.docx]

Supplementary Material

# Supplementary Data

supplemental_biomarkers_table.csv contains taxonomic classifications of all ASVs identified as biomarkers in this study, which groups they were affiliated with, and DESeq2 output statistics. Biomarker ASVs from both 2013 and 2014 datasets are included and named according to dataset of origin. The block column represents the soil microsite number, while crop corresponds to cultivar type.

## Supplementary Figures

Relative abundances of the 100 and 1000 most abundant ASVs within communities are shown in 2013 (Fig. S1) and 2014 (Fig. S2) datasets are identified. Alpha diversity patterns are showed in Fig. S3.


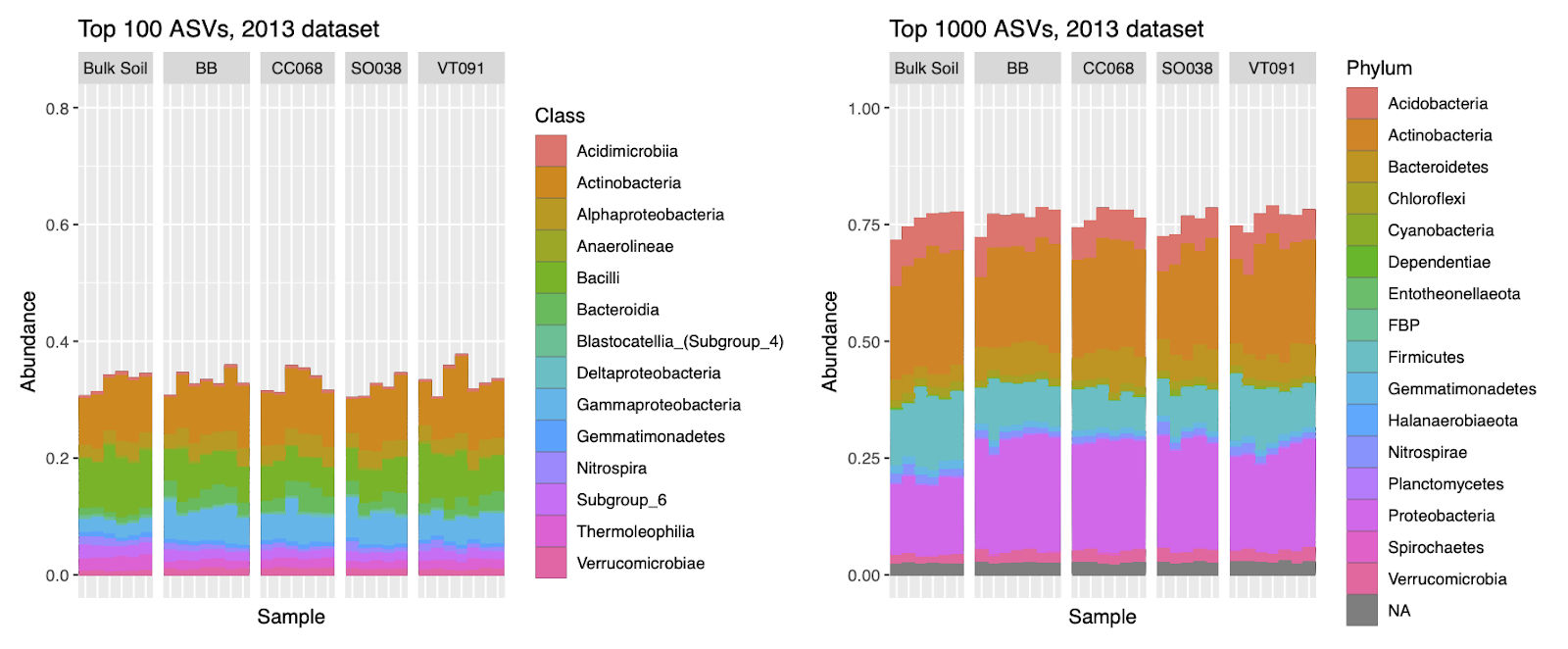


**Figure S1. Soil and rhizosphere bacterial community composition in the 2013 dataset.** Relative abundances show the 100 most abundant ASVs colored by Class (left panel) and the 1000 most abundant ASVs by Phylum (right panel). Vertical panels of rhizosphere communities are titled by bulk soil or *B. rapa* genotype.


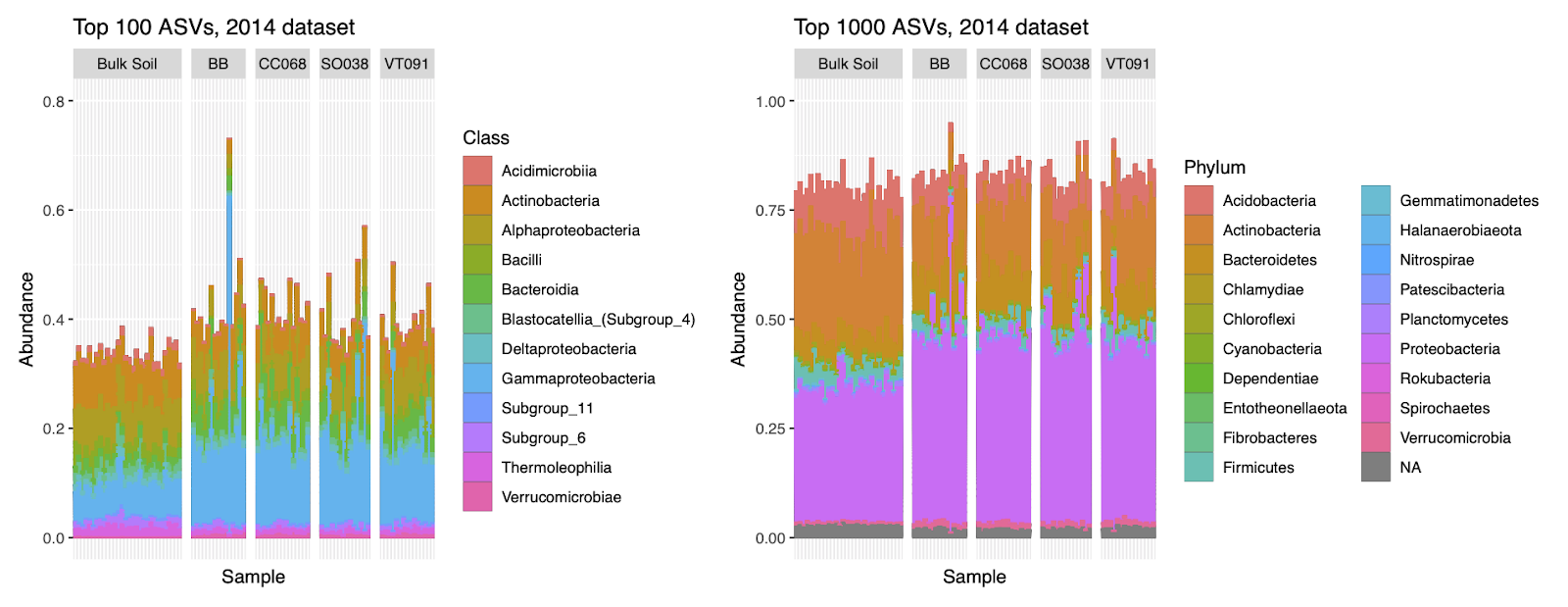


**Figure S2. Soil and rhizosphere bacterial community composition in the 2014 dataset.** Relative abundances show the 100 most abundant ASVs colored by Class (left panel) and the 1000 most abundant ASVs by Phylum (right panel). Vertical panels of rhizosphere communities are titled by bulk soil or *B. rapa* genotype.


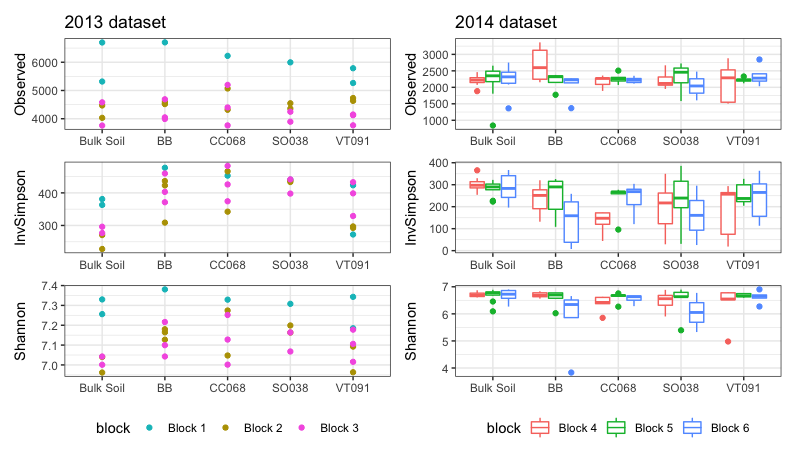


**Figure S3. Alpha diversity of soil and rhizosphere bacterial communities in both 2013 and 2014 datasets.** Values for ASV richness (Observed), Inverse Simpson, and Shannon indices are shown. Rhizosphere communities are grouped by *B. rapa* genotype on the x axis, and boxplots are colored by block. Midpoints of boxplots are medians, and the top and bottom bounds are third and first quartile ranges, respectively. Upper/lower whiskers extend to values no larger/smaller than 1.5 times the range between the first and third quartiles. Groups from 2013 have too few points to be represented as boxplots.

As in the 2014 dataset (Figure 2D within the main text), rhizosphere biomarkers in 2013 make up a higher percentage of rhizosphere communities than do soil biomarkers within soil communities (Fig. S4). In terms of abundance, most rhizosphere biomarkers were consistent between both years, while most soil biomarkers were not. Low but variable percentages of block biomarkers in soil and rhizosphere communities recall patterns similar to the 2014 dataset (Figures 3E and 4D). CC068 and SO038 show higher numbers and percentages of cultivar-specific biomarkers than the others, as in the 2014 dataset (Figure 6D).


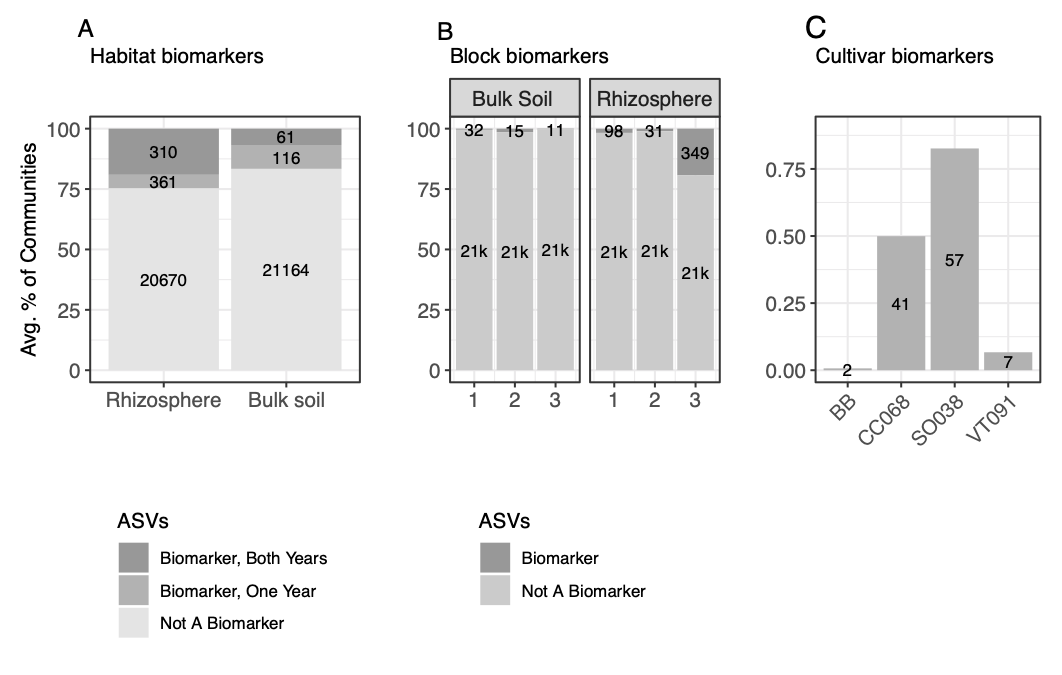


**Figure S4. Biomarker percent abundance contributions to communities in the 2013 dataset.** (A) Numbers and average percent abundances of biomarker ASVs for rhizospheres and soils. Sets of biomarkers for these groups found in both 2013 and 2014 datasets are colored in darkest gray. (B) Total numbers and average percent abundances of biomarker ASVs specific to each block in soil and rhizosphere communities. (C) Total numbers and percent abundances of biomarkers specific to each cultivar.


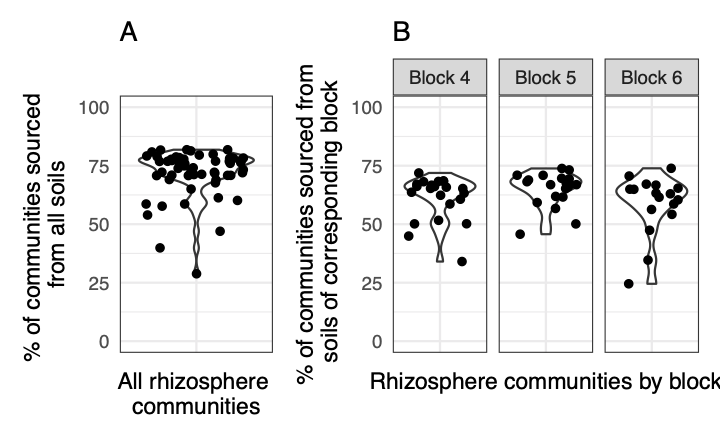


**Figure S5. Distribution of the percentages of rhizosphere communities sourced from respective soils as determined by FEAST.** All rhizosphere and soil communities from 2014 are considered in (A). Source percentages of rhizosphere communities from 2014 according to soil communities from the same block are depicted in (B).

## Supplemental Tables

| genotype | CGN, source country | Description |
| --- | --- | --- |
| BB | Upper Newport Harbor, California, USA | Back Bay, weedy population (Franks et al., 2007) |
| SO038 | CGN06840, Germany | Summer oilseed |
| CC068 | CGN07196, Bulgaria | Chinese cabbage |
| VT091 | CGN10999, United Kingdom | Vegetable turnip |

Supplemental Table 1. Accession numbers and sources of all cultivars. Cultivars were used in both 2013 and 2014 experiments.

| **Measurement** | **Value** |
| --- | --- |
| pH | 7.9 |
| salts (mmhos/cm) | 0.5 |
| Excess Lime | High |
| Texture Estimate | Sandy Clay Loam |
| Organic Matter (%) | 2.1 |
| Nitrate (ppm) | 7 |
| Phosphorus (ppm) | 10.1 |
| Potassium (ppm) | 171 |
| Zinc (ppm) | 0.6 |
| Iron (ppm) | 15.9 |
| Manganese (ppm) | 2.2 |
| Copper (ppm) | 2.5 |
| Boron (ppm) | 0.01 |
| Sulfur (ppm) | 13.4 |

Supplemental Table 2. Physical and chemical characteristics from one field soil sample collected in the 2014 growing season.

## Supplemental References

Franks, Steven J., Sheina Sim, and Arthur E. Weis. “Rapid Evolution of Flowering Time by an Annual Plant in Response to a Climate Fluctuation.” *Proceedings of the National Academy of Sciences* 104, no. 4 (January 23, 2007): 1278–82.<https://doi.org/10.1073/pnas.0608379104>.
